# Effects of Transcranial Direct Current Stimulation on effort during a working-memory task

**DOI:** 10.1038/s41598-021-95639-7

**Published:** 2021-08-12

**Authors:** David Framorando, Tianlan Cai, Yi Wang, Alan J. Pegna

**Affiliations:** School of Psychology, The University of Queensland, Saint Lucia, Brisbane, QLD-4068 Australia

**Keywords:** Psychology, Physiology, Motivation

## Abstract

Transcranial Direct Current Stimulation (tDCS) has shown that stimulation of Dorsolateral Prefrontal Cortex (DLPFC) facilitates task performance in working-memory tasks. However, little is known about its potential effects on effort. This study examined whether tDCS affects effort during a working-memory task. Participants received anodal, cathodal and sham stimulation over DLPFC across three sessions before carrying out a 2-back task. During the task, effort-related cardiovascular measures were recorded—especially the Initial Systolic Time Interval (ISTI). Results showed that anodal stimulation produced a shorter ISTI, indicating a greater effort compared to cathodal and sham conditions, where effort was lower. These findings demonstrate that anodal stimulation helps participants to maintain engagement in a highly demanding task (by increasing task mastery), without which they would otherwise disengage. This study is the first to show that tDCS impacts the extent of effort engaged by individuals during a difficult task.

## Introduction

### Motivation intensity theory

Motivation intensity theory defines effort as the investment of resources to allow the execution of behaviors and its function is to sustain the activity that is needed to attain a goal^1^. This theory is based on a resource conservation principle which posits that people aim to attain their goals using the least possible resources: individuals should perform a task, by mobilizing just the amount of resources that are necessary to succeed, but not more. To do this, individuals need an indicator of the amount of resources required for successful task execution. According to Brehm & Self^1^, this information is provided by task difficulty: the amount of resources required for task accomplishment should increase with task difficulty. However, investing resources to succeed in tasks that are impossible or that are not worth the effort is a waste of resources. Thus, motivation intensity theory assumes that effort should rise with task demand as long as the effort is justified, and the task is feasible^1^. Richter et al.^2^ illustrated this principle by demonstrating that effort increases with task difficulty but drops once the task becomes extremely difficult. In these contexts, when participants grasp that the effort is no longer justified, or that the task is not feasible, they disengage rather than continue to waste effort.

Over these last two decades, a vast amount of research has illustrated the significance of this effect by modifying the perception of task difficulty using different methods. For instance, moods, implicit affects, and even environmental contexts such as light, have all been shown to modify the perception of subjective demand^3–5^, which in turn determines effort. Interestingly, investigations have also found that a person’s self-perception has an important influence on the amount of effort that will be mobilized in a task because of its effect on subjective demand^6,7^. Indeed, individuals with low perceived self-ability will perceive a challenge as being much greater than individuals who possess a high perceived self-ability^8^. Since effort increases with task difficulty (again when possible and justified), individuals with a lower perceived ability should consequently mobilize more effort than individuals with high perceived ability in easy tasks. By contrast, the opposite is true for difficult tasks because individuals perceiving their ability as low, disengage under more difficult conditions, resulting in low effort.

This was illustrated by Wright and Dill^6^ who manipulated the perception of the participants’ ability through feedback delivered to them after they accomplished a first task. Half of them received the information that they had performed poorly compared to others, whereas the other half were informed that they performed well. Subsequently, participants performed a second task, in which difficulty was established as a criterion that participants had to reach to succeed in the task: high (to perform at or above the 85th percentile of previous subjects) vs. low (to perform at or above the 15th percentile of previous subjects). This set the objective task difficulty. Participants who received feedback indicating high ability produced a greater effort in the high compared to the low objective task demand. By contrast, participants who received feedback indicating that they had a low ability mobilized greater effort in the low compared to the high objective task difficulty. This demonstrates that participants receiving low feedback ability disengage when task difficulty is high due to excessive demand, whereas participants with high ability stay engaged (task is difficult but doable).

### Measuring effort

Wright^9^ integrated motivational intensity theory^1^ with the active coping approach^10^. According to Wright (1996), effort should be reflected by beta-adrenergic sympathetic nervous activity on the heart. Beta-adrenergic activity has an influence on cardiac contractility—which can be measured with the pre-ejection period (PEP)—defined as the time interval between the onset of the ventricular depolarization and the opening of the aortic valve^11^. The integration theory of Wright found strong empirical support—evidence reported that PEP sensitively responds to manipulations of task demand^2^, incentive^12^, and a combination of both^5^.

A vast amount of studies still report SBP as a measure of effort^13,14^ due to the systematic impact of cardiac contractility on cardiac output (the volume of blood pumped by the ventricles per minute). However, SBP—and to an even stronger degree diastolic blood pressure (DBP)—is also influenced by peripheral vascular resistance, which is not systematically influenced by beta-adrenergic activation^15^. In addition, other studies still relied on heart rate (HR) to measure effort^16^. However, measures of systolic time intervals such as PEP or the related Initial Systolic Time Interval (ISTI), which produces a variable that is strongly correlated to PEP^17–19^, are more reliable measures of effort, as they directly mirror beta-adrenergic sympathetic impact^20^. Besides, PEP should always be assessed with HR and blood pressure to control for possible preload (ventricular filling) or afterload (arterial pressure) effects on PEP^21^.

### Dorsolateral Prefrontal Cortex

The Dorsolateral Prefrontal Cortex (DLPFC) is part of the prefrontal cortex, which is subdivided into the dorsolateral (i.e., DLPFC), medial (anterior cingulate), and orbitofrontal cortex regions^22^. The literature suggests that the medial and orbital areas are more involved in the emotional/motivational aspects whereas the dorsolateral regions are involved in more cognitive/metacognitive aspects^22^. More specifically, DLPFC has been shown to be particularly involved in executive and complex behaviours and the literature endorses that it plays a role in (1) the manipulation of information in working memory and in (2) the planification processes. Whereas the different contributions of left versus. right DLPFC has remained controversial^23^ and even proposed to be gender based^24^, some evidence tends to suggest the left DLPFC contributes more to working memory process^25^, whereas the right DLPFC is more involved in process beyond the scope of working memory such as goal-directed behavior or decision making^26^.

### Transcranial Direct Current Stimulation

These last decades a vast amount of research attempted to find methods to increase performance of individuals, with a particular emphasis on executive functions^27,28^. One of the neuromodulation methods that has received particular attention is Transcranial Direct Current Stimulation (tDCS). Two electrodes are placed on the scalp on a specific region of interest. Subsequently, a weak electrical current is run from the positive charged cathode to the negative charged anode, and cortical excitability is seen to increase close to the anodal electrode, while a decrease is seen over cathodal stimulation^29^. Stimulation of the dorsolateral prefrontal cortex (DLPFC) has been reported to enhance performance during working memory tasks^30,31^. Interestingly, the effects of tDCS on performance appears to be sustained once the stimulation has ended^31^. Research on tDCS is currently receiving much attention, with studies investigating motor rehabilitation, motor learning^32–34^ and cognitive trainings in older adults^35^, for instance.

Research on tDCS reported that anodal stimulation is an effective way to improve task mastery of participants in mental activities related to the function of the stimulated area. Interestingly, according to research on self-efficacy beliefs, task mastery should influence how individuals perceive their own ability^8^. Bearing in mind that perceived ability should impact effort through its impact on task demand^6^, we hypothesized that anodal stimulation would modify the perception of task demand, resulting in alterations of effort compared to situations where there is no effective stimulation. Anodal stimulation should increase task mastery and consequently participants’ ability to perform the task, resulting in a decreased perception of the subjective task demand. As effort follows task demand, we expected different levels of effort-related cardiovascular responses. To test this hypothesis, we applied tDCS over the DLPFC before participants performed a challenging N-Back task. Participants received anodal, cathodal and sham stimulation throughout three sessions.

### The present experiment

In the present experiment, we therefore examined the effect of tDCS on mental effort. Participants performed three sessions, one for each stimulation condition: anodal, cathodal and sham. During the sessions, stimulation was applied over the DLPFC and was followed by a 2-back mental concentration task while participants’ cardiovascular measures were recorded. We expected that anodal stimulation should improve task mastery in a working memory task, resulting in alterations of subjective task demand and effort.

## Results

### Cardiovascular baselines

To determine stable cardiovascular baseline values and check for any significant differences at baseline, we ran repeated-measures ANOVAs of the 8 1-min scores of ISTI, HR, SBP, and DBP with the three stimulation conditions included as a factor. The 8 × 3 repeated-measures ANOVAs for the ISTI, HR and DBP revealed no significant main effect nor interaction, *Fs*(14, 182) < 1.52, *ps* > 0.105, η^2^ > 0.09. For the SBP, the effect of time was significant, *Fs*(2, 26) < 4.32, *ps* > 0.001, η^2^ > 0.25. Tukey HSD tests revealed that the 7 last minutes did not significantly differ (*ps* > 0.13). Given the absence of significant difference of the baseline measures for ISTI, HR and DBP across time and conditions and the absence of significant difference for the last seven minutes of the SBP, unique scores by indices were created using the last 7 measures of the baselines. Cell means and standard errors appear in Table 1. In addition, no significant effects emerged for the baselines of the other cardiovascular indices (*ps* > 0.229).

**Table 1:** Means and standard errors (in parentheses) baseline values of initial systolic time interval, systolic blood pressure, diastolic blood pressure and heart rate.

	Baseline values
ISTI	137.58 (4.15)
SBP	101.43 (2.06)
DBP	71.24 (2.62)
HR	61.07 (1.24)

*ISTI* initial systolic time interval (in ms), *SBP* systolic blood pressure (in mmHg), *DBP* diastolic blood pressure (in mmHg), *HR* heart rate (in beats/min).

### ISTI reactivity

**Figure 1:**
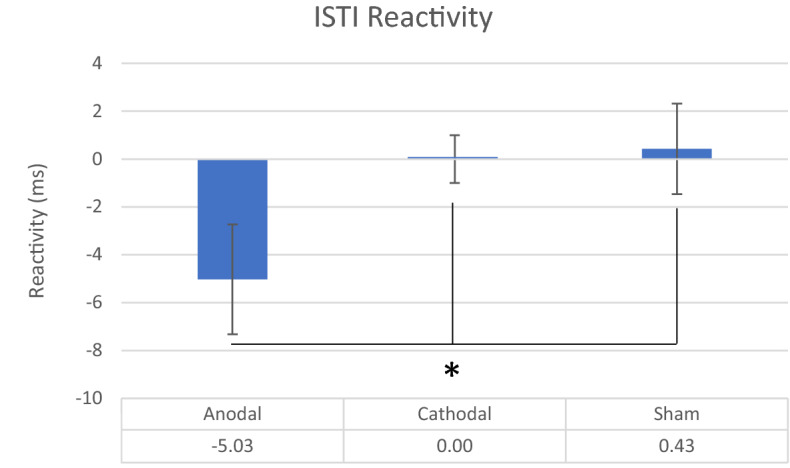
ISTI reactivity (in ms) for the anodal, cathodal and sham stimulation conditions.

The results revealed that the a priori contrast for ISTI reactivity was significant, *F*(1, 16) = 4.59, *p* = 0.047, η^2^ = 0.22. As depicted in Fig. 1, anodal stimulation produced a stronger ISTI (M = − 5.09, SE = 2.29) compared to both cathodal (M = 0.00, SE = 0.99) and sham (M = 0.43, SE = 1.90) conditions.

### SBP, DBP, HR

The a priori contrasts for SBP, DBP and HR were non-significant, *Fs*(1, 16) > 0.94, *p*s > 0.34, η^2^ < 0.06. However, as depicted in Table 2, mean values for all cardiovascular indices follow the pattern of the ISTI.

**Table 2:** Means and standard errors (in parentheses) of systolic blood pressure, diastolic blood pressure and heart rate reactivity scores during the five minutes of task performance.

	Stimulation		
	Anodal stimulation	Cathodal stimulation	Sham stimulation
SBP	3.85 (1.83)	3.31 (1.08)	0.93 (1.61)
DBP	2.44 (0.95)	3.20 (1.03)	1.74 (0.88)
HR	5.10 (2.07)	2.40 (1.44)	3.53 (1.42)

Reactivity scores are calculated by subtracting mean baseline values to mean average task values.

*SBP* systolic blood pressure (in mmHg), *DBP* diastolic blood pressure (in mmHg), *HR* heart rate (in beats/min).

### Task performance and verbal measures

The a priori contrasts for the reaction times and the response accuracy were non-significant, *Fs*(1, 16) > 0.21, *p*s > 0.654, η^2^ < 0.012. Overall accuracy was 78% of correct responses.

The a priori contrasts for the task difficulty, success importance, feeling of ability and perceived performance (mean and standard error are depicted in Table 3) during the task were non-significant, *Fs*(1, 16) > 4.09, *p*s > 0.06, η^2^ < 0.20. The overall rating of the task demand was of 4.93 on a 7 Likert scale.

**Table 3:** Means and standard errors (in parentheses) of task performance and verbal measures.

	Stimulation		
	Anodal stimulation	Cathodal stimulation	Sham stimulation
Accuracy	0.77 (0.02)	0.81 (0.02)	0.75 (0.03)
Reaction times	201.66 (18.79)	199.29 (13.37)	186.62 (14.55)
Task difficulty	5.13 (0.33)	5 (0.26)	4.88 (0.27)
Success importance	4.81 (0.28)	4.5 (0.34)	4.44 (0.026)
Perceived performance	3.81 (0.28)	3.56 (0.26)	3.56 (0.30)
Feeling of ability	4.06 (0.25)	3.94 (0.35)	4.25 (0.35)

Task difficulty, success importance, perceived performance, feeling of ability were measured with Likert scales ranging from 1 (not at all) to 7 (very much).

## Discussion

The present study provides the first evidence of an impact of tDCS on effort-related cardiovascular measures. As hypothesized, participants showed a modification of ISTI in the anodal stimulation condition compared to the cathodal and sham stimulation conditions. This suggests changes in levels of effort^18^. Specifically, anodal stimulation of the DLPFC is associated with shorter ISTI, which is interpreted as the result of higher effort. By contrast cathodal and sham stimulation conditions resulted in extremely low (close to zero) ISTI reactivity, which suggests disengagement.

The link between initial systolic time intervals such as Pre-Ejection Period (PEP) or ISTI and effort has been largely demonstrated^2,9,14,18,19^. Most of the previous research in this area used PEP as an index of resources mobilization (effort), based on the work of Wright^9^, who integrated the active coping approach with the motivation intensity theory^1^. Obrist^10^ reported that the sympathetic nervous system responds to task engagement when individuals have a control over the task outcome when performing well (defined as active coping by this author). Following on this, Wright suggested that sympathetic activity on the heart reflects increased resources mobilization (effort) in active coping contexts. Importantly, the quality of a cardiovascular measures as an index of effort depends on how pure the cardiovascular measure is to assess sympathetic activity on the heart. The idea is that the purest measure of sympathetic activity would be measures that strongly depend on myocardial sympathetic activity. Importantly, systolic time intervals such as PEP or ISTI are considered as one of the purest measures of effort because they directly mirror beta-adrenergic sympathetic impact on heart^20^. This could also explain the reason why only the ISTI showed a significant a priori contrast among the other cardiovascular measures although the pattern of the SBP, HR and DBP corresponds to that of ISTI. Importantly, ISTI effects were not accompanied by decreases in blood pressure or HR, which means that the reported effects on ISTI cannot be explained by pre- or afterload effects^36^. In the current study, we decided to opt for Initial Systolic Time Interval (ISTI) as a measure of effort because it is less prone to errors, relying on salient waveform points that are easily and reliably identifiable. Further, ISTI has been shown to covary strongly with PEP reactivity^18^. ISTI is thus a powerful marker of effort.

Our present hypothesis was that increased mastery of the task would decrease the perception of subjective demand^6^ through its effect on ability beliefs^8^. Confirming this, under anodal stimulation, an increase in ISTI reactivity was observed suggesting that the task was associated with a higher effort, likely due to the fact that although task demand was high, it was perceived as still feasible. By contrast, in cathodal and sham conditions, the reactivity scores were very low, indicative of disengagement, and likely due to excessive task demand as suggested by previous research^5,37^.

Our results are consistent with previous investigations on ability beliefs and effort^6,7^, which previously reported that—in high difficulty contexts—participants with high perceived self-ability show increased levels of effort compared with participants who present low perceived self-ability. Wright reported that subjective demand decreases with ability perception: the lower the ability, the higher the perception of the task demand. Following this principle, increasing subjective task demand in a difficult context (low ability participant) leads to disengagement in order to conserve resources. By contrast, decreasing the perception of task demand does the opposite: the task is hard but doable, resulting in high effort. Correspondingly, our present experiment showed that higher perceived ability in a particular task, induced through anodal stimulation^8^, allows participants to stay engaged in a difficult task. To the best of our knowledge, this study is the first to report an effect of tDCS on the effort-related cardiovascular response. The present results contribute to the understanding of the relationship between task mastery, perceived ability and effort.

However, some authors have reported that DLPFC could be also associated with the significance of success^38,39^. For instance, studies in fMRI reported that DLPFC activation was associated with the presence of external rewards such as monetary incentives^38^ and with internal rewards—manipulated with the notion of task interest^39^. In such contexts DLPFC was activated when importance to succeed in the task was high. According to motivation intensity theory, the value of success determines potential motivation, which in turn establishes how much effort one is willing to exert before disengaging^14^. One might then argue that anodal stimulation over DLPFC increased effort simply because a higher amount of effort was justified: if anodal stimulation over the DLPFC increased the importance of success, participants could have perceived the mobilization of resources as still worthy despite the more challenging task. By contrast, in both cathodal and sham stimulation conditions, the amount of effort justified to succeed in the task could have remained unchanged. Consequently, participants could have disengaged (decrease in effort) because the effort required to succeed in the task exceeded the amount they were willing to apply. Nevertheless, it is not clear whether stimulation over the DLPFC in the absence of internal or external rewards could induce shifts in importance of success. As a matter of fact, other studies reported that stimulation over the DLPFC induced rather a modification in sensitivity to rewards in presence of incentives^21,40^. This in turn, could affect the importance of succeeding in the task due to the fact that the reward is strongly desired. For instance, stimulation of the DLPFC increased acceptance rate in an ultimatum game (in which a proposer and a responder must agree on the division of an amount of money, which could be fair or unfair) even if the offers were unfair and the monetary gains small^21^. Overall, this suggests that anodal stimulation over the DLPFC could modify importance of success in situations where external or internal rewards are present. However, given the absence of such incentives in the present experiment, it appears unlikely that tDCS affected effort by acting on the importance of success. Nevertheless, the differential perspective regarding the role of DLPFC is noteworthy and requires further research.

In our study, we used an offline tDCS design -effects of tDCS were measured after the tDCS system was turned off- to avoid measuring electrical activity on the ECG, which is extremely sensitive to electrical noise. The delay between the stimulation and the actual task may be a source of concern as the initial effects of tDCS were reported using online tDCS designs^30,41^. However, according to recent studies the effects of tDCS on DLPFC appear to be also effective in offline designs—where measures are taken once the stimulation is turned off^42^. For example, studies reported that effects of tDCS on the motor cortex can persist up to 1 h after termination of stimulation^43^. More relevant to our study, Ohn et al.^31^ reported that, DLPFC stimulation effect on a N-back task was still observed 30 min poststimulation. In the present experiment tDCS effects were measured 10 min poststimulation. Thus, the effects observed in our study are within the duration times where effects on performance are reported^11,42^.

In the current experiment, no significant effect was observed on reaction times and response accuracy. Previous studies on effort reported variable effects, with some noting effort-related physiological effects on task performance^44,45^ while others did not^37,46,47^. This may be due to the fact that performance depends on more than effort alone, and that a number of strategies can be used to perform a task^48^. Indeed, some participants may prefer to favor speed over accuracy, while others might prefer to focus on accuracy at the expense of speed. Yet, the absence of significant results on task performance appears to be in contradiction with previous results reporting that anodal stimulation improved accuracy^30,31^. However, the design of the present experiment was determined to maximize the effects of stimulation on effort and not on task performance. Previous experiments that tested tDCS effect on a working memory task used a 3-back task and provided participants with the opportunity to practice for 20 min or until they reached a plateau, before starting. However, a long practice period likely allows task demands to be appraised too clearly, and thus be less easily influenced by stimulation. For this reason, a 2-back task with a noticeably short practice period was used here, to maximize effects of tDCS on perception of subjective task demand, and consequently on effort. These differences in experimental design may well have affected the effects of stimulation on task performance.

It is noteworthy that verbal measures recorded at the end of the task were non-significant. More specifically, the rating of the task demand, the importance of success, the perceived self-ability, and the perceived performance did not show any significant effect. The lack of effects on self-reported measures is in line with previous research on effort. In several studies, experimental manipulations that were intended to affect effort modified cardiovascular measures, but no effects were reported on verbal measures such as post-task difficulty^3,44^. It appears that the nature of the mechanisms reflecting changes in levels of effort are either not fully conscious and are not easily measurable with self-report measures^49^ or are hardly measurable at the end of the task—especially with a one question item. Indeed, according to Tourangeau^50^, feelings experienced during the task (conscious or not) suffer from a memory biases—rendering post-task questions unreliable. Overall, this also suggests that the stimulation’s effect in the context of effort—through task mastery and perceived ability—is likely not a fully conscious process.

## Limitations

In the present experiment, tDCS was administered through a conventional montage. Recent discussions have risen about the diffusion of the current with such techniques^51^: it has been argued that it is difficult to ascertain whether stimulation is the strongest at a specific site. Indeed, the current applied is often not focalised on the target site but diffuses to several areas^52^. One of the problems that has been identified is for instance the use of large electrodes, which creates a widespread change in cortical excitability around the target area^53^. One solution for future research would thus be the use of high-definition Transcranial Direct Current Stimulation (HD-tDCS), which has been recently developed to overcome current distribution problems. This technique uses several return electrodes and allows more focused brain modulation^54^.

Some research has also reported that stimulation of the DLPFC might not stimulate solely the DLPFC, but also remote regions beneath this area. For instance, recent studies reported that tDCS over left DLPFC modulated the cortical activity of striatal regions^55^, which are regions associated with motivational processes^56^. Although to our knowledge there is no evidence for the role of the striatum in effort context, we cannot completely exclude the potential effect of tDCS on striatal regions. If the stimulation of such areas were to affect motivation, they would also potentially affect effort in contexts where motivation exerts a visible effect on effort (e.g., when a task becomes exceedingly difficult). In this context, an increase in motivation would give rise to higher levels of effort and prevent participants from disengaging, which corresponds to our current observations in the anodal stimulation condition. Further studies would be required to disentangle this eventuality.

## Conclusion

In summary, the present experiment showed that anodal stimulation of DLPFC modulates effort in a difficult working memory task. Through its implicit effects on perception of self-ability, anodal stimulation appears to maintain participants engaged in the task by producing a feeling of feasibility in the face of difficulty. By contrast, under cathodal and sham stimulations conditions, this is not observed suggesting that the task appears otherwise too demanding, subsequently leading to disengagement.

## Method

### Subjects and design

All methods were performed in accordance with the guidelines and regulations at The University of Queensland. TheTwenty-two university subjects were recruited and participated in the study to comply with recommendation of Simmons et al.^57^—to collect at least 20 observations per cell. Participants were University of Queensland students who had to be between 18–35 years of age, had to be free from epilepsy, and were required not to wear a pacemaker. Five of these participants were excluded due to loss of signal, 1 due to extremely delay between sessions (COVID-19 restrictions), 1 due to data loss, and 3 due to excessively poor accuracy (< = 50%). The final sample was therefore composed of 17 subjects (10 females). The mean age of the sample was 21.82 years (SD = 0.47 years; age range 19–27). All subjects gave their informed written consent, passed a tDCS safety-screening questionnaire, and were paid for their time. The study was approved by the University of Queensland Human Research Ethics Committee. Participants were asked to attend three different sessions separated by a 1-week interval—one for each stimulation: Anodal, Cathodal and Sham conditions. For each session, effort-related cardiovascular measures were recorded. The order in which the types of stimulation were delivered was fully counterbalanced across participants. ISTI, SBP, DBP and HR were measured during the two periods composing each session: the habituation (rest) period and the task execution period.

### Cardiovascular measures

**Figure 2:**
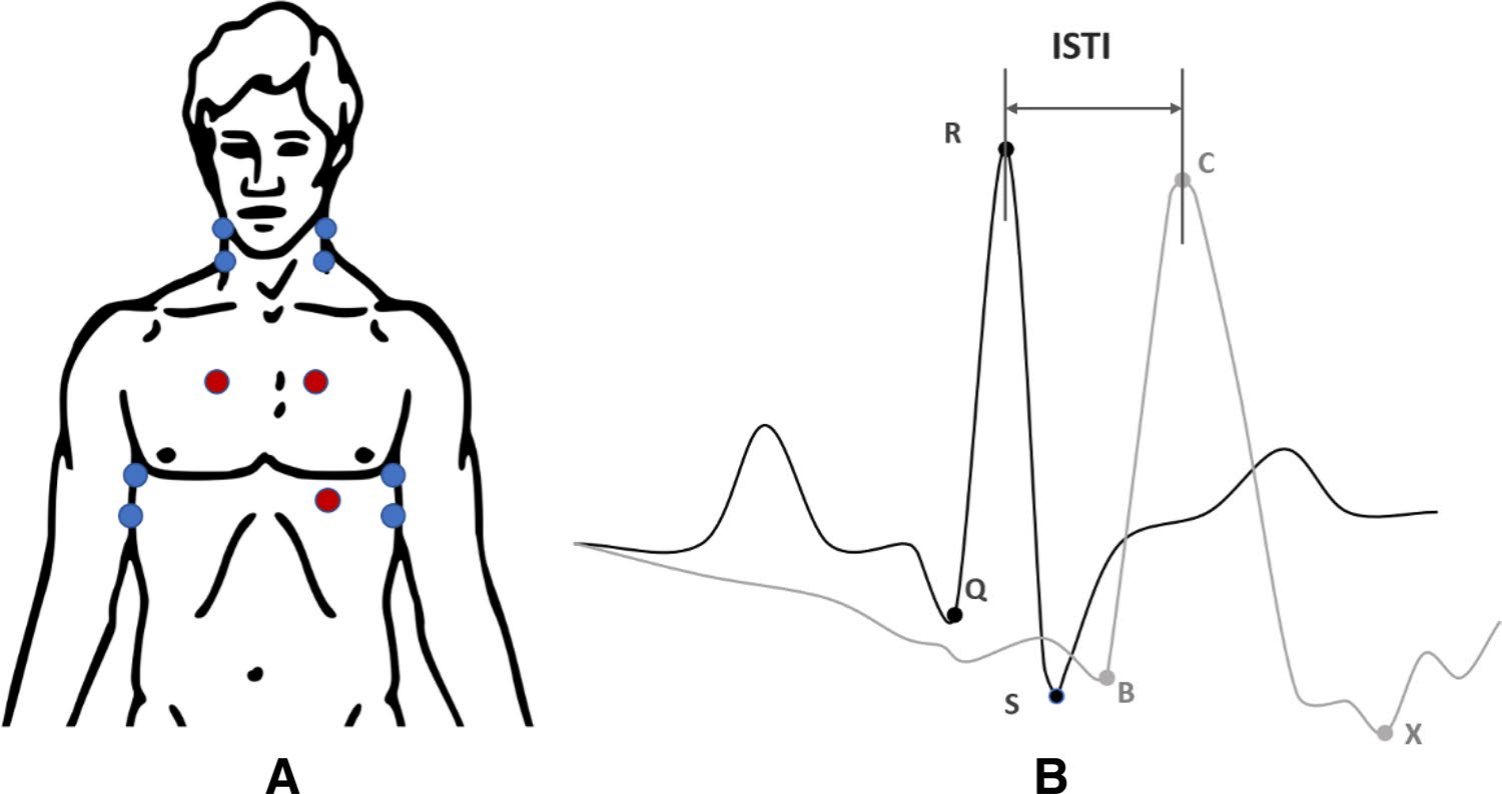
(**A**) The positions of ECG (in red) and ICG (in blue) electrodes. (**B**) The ECG (dark grey) and ICG signals (light gray). The Initial Systolic Time Interval (ISTI) is calculated on the time interval between the R-wave peak (ECG) and the peak of the dZ/dt wave (ICG)-C point.

ISTI (in ms) was calculated using an impedance cardiogram (ICG) and electrocardiogram (ECG) signals measured with a Biopac MP150 system (BioPac systems Inc., Santa Barbara, CA). ICG electrodes were placed on participants’ neck, on each side of the middle axillary line at the height of the xiphoid (with two on each side). ECG electrodes were placed below the clavicles, on each side of the frame of rib cage and on the left side of participants’ pectoral muscle, at lower edge of left rib cage (see Fig. 2A). Signals were transferred to digital data (sampling rate 1000 Hz) and analysed (50 Hz low pass filter) offline using the Acknowledge software (V 4.1.0-150 M, BIOPAC Systems, Inc.). To assess ISTI, we measured the time interval between the R-wave peak and the peak of the dZ/dt wave-C points ^30,31,41^. The QRS points in heart rate and the C points in blood flow were located using the software and manual correction. ISTI was determined as the interval between the QRS point and the C point (see Fig. 2B).

### tDCS

Stimulation was administered with a NeuroConn stimulator, using 5 × 5 cm saline-soaked pair of surface sponge electrodes. The location of the target electrodes was determined using the 10–20 EEG system^58^. To stimulate the DLPFC, the anodal electrode was placed over F3 whereas the cathodal electrode on the contralateral right area. This method of localizing DLPFC has been used in past TMS studies^59^, has been shown to be accurate according to neuronavigation techniques^60^, and has been widely used by studies investigating DLPFC stimulation on working memory performance^30,41^. Stimulation lasted 11 min in total including 30 s ramp on and 30 s ramp off time for the current. For the sham stimulation condition, the current was administered for 75 s. The current density for the anodal and cathodal conditions was 1 mA. The current density for the sham condition was 0.7 mA. Participants were unaware of the type of stimulation.

### Procedure

Participants were seated in a comfortable chair in front of a 60 Hz refresh rate monitor, were given signed consent, and were equipped with tDCS and physiological sensors. Then, the computer program running the experiment (E‐Prime, Psychology Software Tools, Pittsburgh) started and the experimenter went to another location in the room which was separated by a black curtain. Participants started by answering biographical questions (age, gender, etc.…). This was followed by a hedonically neutral movie (10 min) while tDCS was administered (anodal, cathodal or sham stimulation). After the stimulation period, the tDCS stimulation was stopped and a second hedonically neutral movie was presented for 8 min while cardiovascular baseline values were recorded. Next, participants received task instructions and performed 40 practice trials of a version of a 2-back working memory task. In this task, letters were presented for 100 ms, followed by a blank screen for 2000 ms. Participants were asked to respond as quickly as possible by pressing a green key – the number 4 of the numeric pad—when a letter matched the one that had appeared 2 stimuli before. The task consisted of 160 trials, with 49 trials requiring a response. Task performance was calculated based on the number of correct keys presses divided by the total number of correct response (49). The 2-back task lasted 5 min while physiological measures were recorded. After the task, participants needed to answer several questions (presented at the screen) with the keyboard (1–7). Participants were asked to rate subjective task difficulty (“*How difficult did you find the task?*”), the importance that they attributed to success (“*How important was it for you to get the job done well?*”), how they felt they had performed (“*How well do you think you performed during the task?”)* and how well they felt they had been able to do the task (“*How well did you feel able to do the task?*”) on a Likert scale (1—*not at all;* 7—*very much*).

### Reactivity scores and data analysis

To compute the cardiovascular reactivity scores for the different stimulation conditions, we first calculated baseline scores for the ISTI, SBP, DBP and HR measures (see results section) assessed while participants were at rest (seeing the neutral hedonic movie). Second, we averaged the five 1-min scores of ISTI, SBP, HR, and DBP assessed during task performance for each stimulation condition—anodal, cathodal, sham—and subtracted the baseline values. Our hypotheses were tested through a priori contrasts (anodal + 2; cathodal − 1; sham − 1).
